# Editorial

**Published:** 1864-11

**Authors:** 


RICHMOND, NOVEMBER, 1864.

*AYRES & WADE......PUBLISHERS AND PROPRIETORS.*

### EDITORIAL.

*Prospectus of the Confederate States Medical and Surgical Journal. Second Year.*

Thanks to the influence and support of the Surgeon General and Medical Department of the Army, and to the zealous co-operation of the profession at large, we have the satisfaction of announcing to our friends and readers, that the *Confederate States Medical and Surgical Journal* has, in one year, reached a circulation hitherto unattained by any scientific publication in the South, and in spite of many difficulties and drawbacks, hopes to merit still more the patronage of the Southern medical profession.

Owing to the scarcity and high price of paper, it is absolutely necessary that the publishers should ascertain, as early as practicable, the probable size of their January edition, and hence, all persons intending to subscribe for the year 1865 are earnestly requested to forward their names and subscriptions, either by express, or, if in the army, through the Surgeon General's office, before January 1st. They will be careful to state exactly name and post-office, or army corps to which they are attached.

Subscription for the year 1865, $20, invariably in advance. Express charges will be paid by the publishers, and the postage to army subscribers will be paid at the Richmond office. All communications should be addressed to Messrs. Ayres & Wade, Richmond.

The *Journal* will be under the same editorial management as heretofore, with a large and increasing corps of collaborators. Its communications with Europe will enable the editor to furnish a synopsis of all recent foreign publications of value; and it is believed that the home profession will aid in this effort to found a Southern Medical periodical on a firm basis, by supporting with purse and pen our infant enterprise.

